# The social lives of point-of-care tests in low- and middle-income countries: a meta-ethnography

**DOI:** 10.1093/heapol/czae054

**Published:** 2024-06-22

**Authors:** Janet Perkins, Clare Chandler, Ann Kelly, Alice Street

**Affiliations:** Department of Social Anthropology, School of Social and Political Science, University of Edinburgh, Chrystal Macmillan Building, 15a George Square, Edinburgh EH8 9LD, United Kingdom; Department of Global Health and Development, London School of Hygiene and Tropical Medicine, 15-17 Tavistock Place, London WC1H 9SH, United Kingdom; Department of Global Health and Social Medicine, King’s College London, Bush House North East Wing, 30 Aldwych, London WC2B 4BG, United Kingdom; Department of Social Anthropology, School of Social and Political Science, University of Edinburgh, Chrystal Macmillan Building, 15a George Square, Edinburgh EH8 9LD, United Kingdom

**Keywords:** Point-of-care tests, diagnostics, qualitative evidence synthesis, meta-ethnography, global health innovation, health systems strengthening, health policy

## Abstract

Point-of-care tests (POCTs) have become technological solutions for many global health challenges. This meta-ethnography examines what has been learned about the ‘social lives’ of POCTs from in-depth qualitative research, highlighting key social considerations for policymakers, funders, developers and users in the design, development and deployment of POCTs. We screened qualitative research examining POCTs in low- and middle-income countries and selected 13 papers for synthesis. The findings illuminate five value-based logics—technological autonomy, care, scalability, rapidity and certainty—shaping global health innovation ecosystems and their entanglement with health systems. Our meta-ethnography suggests that POCTs never achieve the technological autonomy often anticipated during design and development processes. Instead, they are both embedded in and constitutive of the dynamic relationships that make up health systems in practice. POCTs are often imagined as caring commodities; however, in use, notions of care inscribed in these devices are constantly negotiated and transformed in relation to multiple understandings of care. POCTs promise to standardize care across scale, yet our analysis indicates nonstandard processes, diagnoses and treatment pathways as essential to ‘fluid technologies’ rather than dangerous aberrations. The rapidity of POCTs is constructed and negotiated within multiple distinct temporal registers, and POCTs operate as temporal objects that can either speed up or slow down experiences of diagnosis and innovation. Finally, while often valued as epistemic tools that can dispel diagnostic uncertainty, these papers demonstrate that POCTs contribute to new forms of uncertainty. Together, these papers point to knowledge practices as multiple, and POCTs as contributing to, rather than reducing, this multiplicity. The values embedded in POCTs are fluid and contested, with important implications for the kind of care these tools can deliver. These findings can contribute to more reflexive approaches to global health innovation, which take into account limitations of established global health logics, and recognize the socio-technical complexity of health systems.

Key messages

- Tracing the ‘social lives’ of point-of-care tests (POCTs) through a meta-ethnographic approach reveals social and temporal intersections between processes of technological innovation and health systems.
- Five value-based logics emerged through this synthesis as central to shaping the life cycle of POCTs: technological autonomy, scalability, care, rapidity and certainty.
- A ‘social lives’ approach and the logics that emerged through this synthesis can contribute to reflexive and sensitive approaches to global health policy-making that understand innovation and health systems as entwined and take into account the fluid values and relationships that shape the life cycle of a product.

## Introduction

In recent years, point-of-care tests (POCTs) that do not rely on modern laboratory equipment and provide rapid results have become a widespread feature of global health programmes. Designed to be mobile and easy-to-use, these small, self-contained, testing devices are often portrayed as a groundbreaking solution to the challenges of delivering laboratory-based testing services in hard-to-reach locations and under-resourced health systems (Peeling and Mabey, 2010; Drain *et al*., 2014; Mitra and Sharma, 2021). A potent symbol of modernity, the laboratory is regarded as a feature of high-income settings—demanding robust infrastructures and systems of transportation, electrification, refrigeration, quality assurance and training in place. The idea of a miniature testing device that has laboratory expertise and infrastructure ‘built-in’ therefore holds considerable allure as a solution to improving access to diagnostics in low- and middle-income countries (LMICs) (Mabey *et al*., 2004; Peeling and Mabey, 2010; Pai *et al*., 2012).

Advocates for the importance of diagnostics to global health goals have lamented the treatment of diagnostic testing as the poor sibling to more glamorous pharmaceutical and vaccine technologies, which have historically been accorded far greater levels of attention and funding at international and national levels (Pai *et al*., 2019). Yet, in recent years the status of diagnostics in these discussions has been elevated, and their technological innovation, development and deployment are now viewed as a priority for global health institutions, programmes and agendas (World Health Organization, 2019; Fleming *et al*., 2021). Over a relatively short period of time, since the early 2000s, we have witnessed the high-profile rollout of POCT devices in response to the human immunodeficiency virus (HIV) epidemic (Pai *et al*., 2014; Armstrong-Mensah *et al*., 2021), as part of renewed efforts to eliminate malaria (Aidoo and Incardona, 2022), to control the spread of drug-resistant tuberculosis (Drain *et al*., 2019) and as a first-line response to emerging disease outbreaks and epidemics, including Ebola, Zika and coronavirus disease 2019 (COVID-19) (Kameda *et al*., 2021; Kelly *et al*., 2022). Diagnostics are now also seen as central to the global response to antimicrobial resistance (Rupasinghe *et al*., 2024). The contribution made by POCTs to the global response to COVID-19, especially in the months prior to the development of a proven vaccine, has significantly raised the profile of diagnostic technologies, prompting calls for the development of tests for a greater number of diseases, the improved availability of World Health Organization (WHO)-identified ‘essential’ tests for routine care at a primary care level and the decentralization of diagnostics to community settings (Fleming *et al*., 2021; Titanji and Pai, 2023).

POCTs have also become objects of curiosity for critical scholars of global health who are interested in understanding global health as a dynamic assemblage of social and cultural norms, institutions and relationships. A substantial body of social science research has focused on these devices since the turn of the century, spanning the fields of science and technology studies, sociology, anthropology and geography (Macdonald and Harper, 2019; Street and Kelly, 2021; Engel *et al*., 2022). This body of critical scholarship has examined the value-based assumptions that underpin the development and use of these devices and the ways in which their introduction has reconfigured health systems, clinical practice and global health security.

Despite this growing corpus of work, few published reviews on POCTs have incorporated qualitative evidence and critical social science scholarship. Existing reviews have largely taken the form of quantitative systematic reviews, focusing on clinical and diagnostic or economic features of specific POCTs (Shivkumar *et al*., 2012; Pham *et al*., 2016b; Casas *et al*., 2018; Figueroa *et al*., 2018; Nacher *et al*., 2018; Verbakel *et al*., 2019; Grillo‐Ardila *et al*., 2020; Van Hecke *et al*., 2020; D’Hulster *et al*., 2022). The few reviews that consider the qualitative social science evidence base tend to engage with this evidence peripherally within the context of a broader systematic review that prioritizes quantitative evidence (Pham *et al*., 2016b; Gliddon *et al*., 2017). Many reviews of qualitative research in this field are carried out unsystematically and employ an aggregative, narrative approach (Kuupiel *et al*., 2017; Rutstein *et al*., 2017; Heidt *et al*., 2020). Others focus on implementation or economic variables related to the implementation of a specific POCT device (Pham *et al*., 2016a; Camara *et al*., 2021; Saweri *et al*., 2021; Engel *et al*., 2022; Martin *et al*., 2022).

The increasing centrality of POCTs to global health policy, interventions and moral imagination in the post-COVID era make this an important moment to take stock of existing contributions from social science to understand POCT technology. Elsewhere, we have described the geographic, disease profile and dimensions of social research on POCTs in recent decades, pointing to areas that have been overlooked and the need for social researchers to be proactive in identifying needs and approaches for social research on these technologies (Perkins *et al*., 2024). In this article, we employ meta-ethnography to examine what has been learned about the ‘social lives’ of POCTs from in-depth qualitative research (Appadurai, 1988; Whyte *et al*., 2002). A social lives approach analyses POCTs as mobile and malleable objects that intersect with multiple people, places, infrastructures and institutions along their life course—material substances that take on different meanings and hold different kinds of value for people as they circulate within processes of commoditization, globalization and localization. As POCTs are not ready-made objects, we also attend to the moments of anticipation, expectation, development, design, regulation and procurement of POCT devices and consider their life course beyond the point of use. Here, we follow biographical approaches from anthropology and science and technology studies that understand values, meanings and relations as being built into the materiality of technological objects rather than simply attached to them. We view those objects as always unfinished entities, whose purpose and value continue to be negotiated and transformed across the full duration of their life course (Kopytoff, 1986; Hyysalo *et al*., 2019).

Through this meta-ethnography, we seek to illuminate core concepts and approaches in the social study of global health technologies, and highlight key social considerations for policymakers, funders, developers and users, in relation to the design, development and deployment of POCTs for purposes of health systems strengthening. Through our analysis, we identify five core value-based logics that underpin current global health innovation frameworks and show how these values and logics are contested, negotiated and transformed within social relationships throughout the POCT life cycle: technological autonomy, scalability, care, rapidity and certainty. We argue that awareness of these logics and their ongoing transformation within social relationships can contribute to a more reflexive approach to the development, deployment and use of POCTs specifically and to global health innovation and policy-making moreJ generally.

## Methods

We carried out a meta-ethnographic synthesis, basing our methodological approach on the well-established meta-ethnographic approach originally proposed by Noblit and Hare (1988) (Noyes *et al*., 2015), which accommodates the systematic comparison and integration of rich conceptual data as well as the engagement of thick qualitative evidence. We were guided by the eMERGe Reporting Guidance for reporting meta-ethnography as proposed by France *et al*. (2019a) and carried out this synthesis following the seven phases suggested by Noblit and Hare (1988). See Table 1 for a summary of these phases.

**Table 1.** Seven phases of the meta-ethnography

Phase 1: Identify the intellectual interest	We identified social dimensions of the development, diffusion and use of POCTs in LMICs, which interact throughout the POCT life cycle as the core intellectual interest of this study.
Phase 2: Decide what is relevant	We determined the following criteria as delineating relevance for inclusion: published, empirical qualitative studies published after 2000; LMICs as context of deployment and use; consideration of *in-vitro* POCTs; consideration of any social phenomena of POCTs throughout the life cycle; and papers providing a rich interpretive account rather than a primarily descriptive account.
Phase 3: Read the studies	We carried out an initial reading of papers to code and extract descriptive information regarding the study, i.e. the objectives, settings, perspectives, and methods, followed by iterative reading and re-reading of papers to extract empirical, conceptual and interpretive data.
Phase 4: Determine how the studies are related	We generated paper-based mind maps to draw out common and recurring concepts across situated studies, mapping out how these concepts were related between papers while maintaining the context of each paper.
Phase 5: Translate the studies into one another	We translated key concepts by comparing them between the different studies and teasing out how they were related and how they differed.
Phase 6: Synthesize the translations	We identified the social dimensions that emerged through the body of papers as a whole, placed the treatment of these dimensions in conversation with the others, and developed a line of argument based on the translation to build ‘make a whole more than the sum of the parts imply’ (Noblit and Hare, 1988).
Phase 7: Express the synthesis	We expressed the synthesis through the write-up of this paper and generated a visual representation (Figure 3).

In the initial stages of the review (Phases 1–3 in Table 1), we identified the social lives of POCTs in LMICs as our area of intellectual interest and carried out an exhaustive literature search and screened the results. From all included studies, we extracted data into an Excel spreadsheet regarding the methodology, study context (e.g. study setting, type of device, health condition and perspective) and social aspects addressed in the paper. All data were extracted by two team members and verified for consistency. Discrepancies were resolved by discussion. In addition, we assessed the richness of the evidence presented in each study, adapting the tool proposed by Ames *et al*. (2019). Our primary adaptation to Ames’s tool was an increased emphasis on interpretive and conceptual work and engagement with social science theory. Studies that demonstrated firm theoretical grounding, strong conceptualization and rich interpretations scored higher in our data-richness assessment (Appendix 2 in Supplementary material). Two reviewers from the research team assessed each study’s data richness, and discrepancies were resolved through discussion. All studies to which we assigned a score of 4 or 5 in data richness were eligible for inclusion in the sampling frame.

From the studies included in the sampling frame (*n* = 24), we selected a review sample for the meta-ethnography synthesis, aiming to represent a broad range of studies relating to the moment(s) of the POCT life cycle represented, the country and region in which the study was carried out and the type of test (format and target) explored (see Table 2). We first categorized the studies by life cycle moment, i.e. whether the focus was on POCT moments of use (sample collection, result interpretation and post-result action) or non-use (research and development, procurement, policy-making, supply-chain management, storage and waste management). As most papers described moments of POCT use, we tried to maximize the inclusion of papers that explored the POCT in other situations. When deciding between papers that focused solely on non-use as opposed to those that provided an account of both moments use and non-use, we prioritized the latter. Thereafter, we considered the geography of the studies and selected studies representing diversity in geography. Finally, we considered the type of test and selected studies to represent the variety of tests explored in the studies included in the sampling frame. All studies included in the sample engaged with social science theory and concepts, and most were explicit in drawing from ethnographic approaches or presented a portion of a longer-term ethnographic engagement with the study setting.

**Table 2.** Descriptive table of included studies

Author, date	Country	Perspective	Setting	Phenomena of interest	Time (moment in POCT life cycle)	Methods
Beckmann *et al*., 2022	Zimbabwe	Formal HIV testers: primary HIV counsellors, nurses, laboratory technicians	14 health facilities: 8 district hospitals and 6 local-level facilities (government, church- and city council-run facilities)	Health staff experiencesand engagement with rapid HIV tests	Sample collection, interpretation of results, post-result action	Rapid ethnographic approach using interviews with health service providers and observations of testing practices
Beisel *et al*., 2016	Sierra Leone, Tanzania, Uganda	Clinicians, laboratory staff, community-level health workers, drug shop retailers, local residents	Lower-level health facilities, community including retail drug shops (Uganda), hospitals and health centres (Tanzania); community service delivery through community health workers (Sierra Leone)	The use of mRDTs in their everyday settings	Supply-chain management, sample collection, result interpretation, post-result action	Interviews with clinical staff and drug shop customers; focus group discussions with local residents, clinicians and drug shop vendors; participant observation in lower-level health facilities and with community-level health workers; ethnographic fieldwork in hospitals and among community-level health workers; discourse analysis of newspaper reports
Umlauf, 2017	Uganda	Health service providers, drug shop vendors, patients	Public health facilities and commercial drug shops	Presumptive treatment of malaria as non-adherence to negative mRDT results	Supply-chain management, sample collection, result interpretation, post-result action	Ethnographic fieldwork in public health facilities
Umlauf and Park, 2018	Uganda	Health service providers	Public hospitals and primary-level facilities	Interruptions in the supply of mRDT in the public health sector	Supply-chain management, sample collection, result interpretation, post-result action	Ethnographic fieldwork in public health facilities
Hutchinson *et al*., 2017b	Tanzania	Health service providers	Public sector clinics (primary level)	The introduction of mRDTs in public health facilities to guide the use of antimalarials	Post-result action (clinical management practices in relation to test results)	Interviews with health workers
Hutchinson *et al*., 2015	Uganda	Drug shop retailers, healthcare providers, patients, local residents	Drug shops	The introduction of mRDTs in local drug shops	Sample collection	Focus group discussions with drug shop vendors, drug shop clients and local residents, and health service providers
Janssen *et al*., 2021	South Africa	Health service providers, patients	Health clinics	The implementation of human immunodeficiency self-testing (HIVST) using a mobile application	Sample collection, result interpretation, post-result action	Ethnographic data generated through observations, interviews and focus group discussions with patients and healthcare workers
Engel *et al*., 2017	India	Patients, health service providers	Community health service delivery settings (through community health workers (CHWs)), public primary-level facility, private clinics	Patients’ trajectories to arrive at diagnosis through POCTs	Sample collection, result interpretation, post-result action	Focus group discussions (embedded in a larger qualitative study involving interviews and site visits)
Engel and Krumeich, 2020	India, North America, Europe	Transnational diagnostic developers, donors, members of civil society, industry consultants, international organizations, policymakers, regulators and researchers; national diagnostic developers, decision-makers, NGO programme officers, scientists, TB and HIV programme officers, laboratory managers, technicians and nurses	Diagnostic companies, funding bodies, transnational regulators, national policy settings, primary-level health facilities	The negotiation of value frames in the development of HIV RDT, TB Xpert diagnostics	Research and development, manufacturing, sample collection, result interpretation	Semi-structured interviews with diagnostic developers, donors, members of civil society, industry consultants, international organizations, policymakers, regulators and researchers; visits to workshops, companies and conferences in Europe and North America; ethnographic fieldwork in Bangalore, India, including 15 interviews with diagnostic developers, decision-makers, NGO programme officers, scientists, TB and HIV programme officers, laboratory managers, technicians and nurses using TB and HIV diagnostics, as well as visits to companies, clinics and laboratories
Haenssgen *et al*., 2018	Thailand	Health service providers, patients	Public primary health facilities	The introduction of CRP-POCT to guide antibiotic prescribing practices	Sample collection, result interpretation, post-result action	Interviews and focus group discussions with healthcare providers and patients
Kelly *et al*., 2022	Brazil, Sierra Leone, Europe, North America	Funders, researchers and developers, manufacturers, industry, transnational policymakers and regulators, national policymakers and regulators	Transnational and national regulatory and policy-making settings, diagnostic companies, funding organizations	The development and regulation of Ebola RDTs, Zika RDTs and COVID-19 RDTs for epidemic response	Research and development, manufacturing, procurement, supply-chain management, sample collections	Qualitative data generated through multiple research projects, including work on the role played by novel diagnostics during the 2014 West Africa Ebola outbreak; a study of experts’ efforts to grapple with scientific uncertainty during the 2015–16 Zika epidemic; and an investigation into the new humanitarian frontiers created by the COVID-19 pandemic; fieldwork carried out in Sierra Leone, Brazil, Switzerland, the UK and the USA; interviews with product developers, research scientists, regulators, government officials and representatives from international health organizations centrally involved in epidemic response; review of policy and scientific literature
Street, 2023	North America (device designed for use in sub-Saharan Africa)	Diagnostic developers	Diagnostic company	The development (and failure of) a CD4 POC device for patients with HIV	Research and development	Case study, interviews, site visits, analysis of media sources and company documents
Engel, 2020	North America, Europe, Africa, India	Funders, researchers and developers, manufacturers, industry, transnational policymakers and regulators, NGOs, formal healthservice providers	Diagnostic companies, funding bodies, transnational and national policy-making settings, hospitals	The development and design of POCTs for TB and HIV	Policy-making, research and development, manufacturing, sample collection, post-result action	Fieldwork and interviews among global health actors involved in diagnostic development for tuberculosis and HIV. These include diagnostic developers, donors, members of civil society, industry consultants, international organizations, policymakers, regulators and researchers

In the synthesis phases of the review (Phases 3–7 in Table 1), we considered the central arguments, theoretical approaches and the meanings of the metaphors, concepts and themes used in each paper to explore how they are related in terms of similarities, differences and their ‘lines of argument’. We translated the studies into one another using paper-based mind maps, connecting how authors frame similar concepts and themes and the language they use to communicate these, paying attention to the assumptions, motivations and ideologies behind each study. The mind maps were then transformed into electronic format using Excel, and this was used as a basis for iterative analysis through a series of team meetings to interpret the data and develop overarching concepts and a line of argument around these.

## Findings

**Figure 1.**
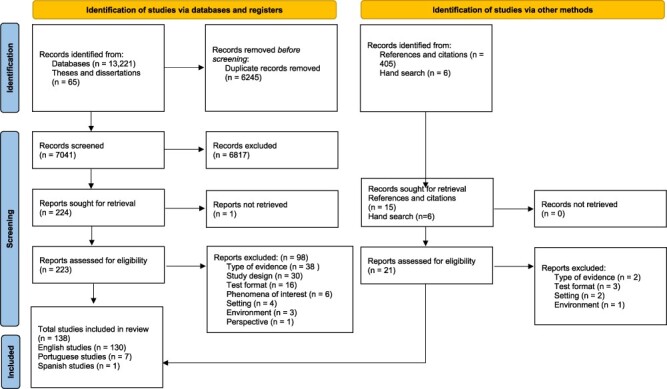
Preferred Reporting Items for Systematic Reviews and Meta-Analyses Preferred Reporting Items for Systematic Reviews and Meta-Analyses (PRISMA) flow diagram depicting the search and screening process

A total of 138 papers were eligible for inclusion from the initial exhaustive search (Figure 1).

**Figure 2.**
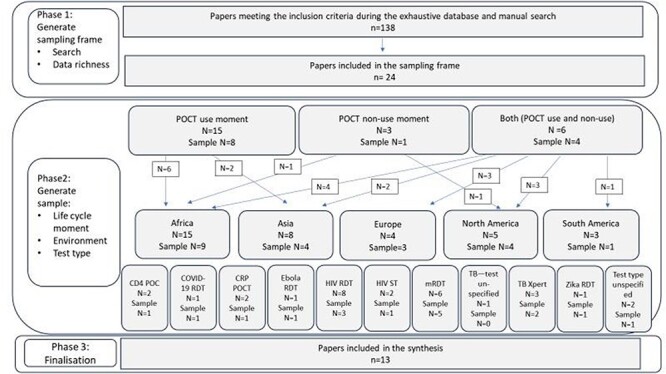
Sampling strategy

Of these, 24 were scored 4–5 in richness and met the criteria for inclusion in the sampling frame. We selected 13 studies from our sampling frame for the synthesis, as summarized in Figure 2.

We carried out a critical appraisal of the included studies based on the tool designed by the Joanna Briggs Institute (Aromataris and Munn, 2020, p. 67). Two members of the review team independently appraised each of the synthesis studies according to these criteria. Any discrepancies were resolved through discussion. All 13 papers reviewed were deemed high quality, likely because they were subject to a preliminary screening and were included in the sampling frame based on their richness.

### The line of argument

**Figure 3.**
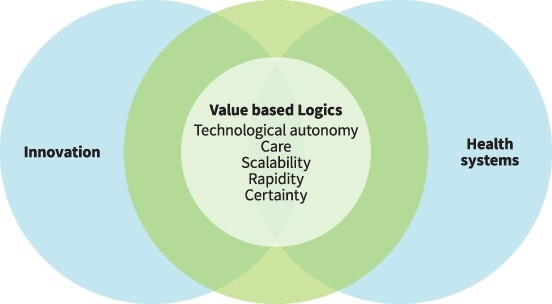
Social lives of POCTs

We found that the social lives of POCTs provide a sharp lens through which to view the values and relationships that underpin the global health innovation ecosystem. Processes of POCT funding, policy, regulation and use reveal the centrality of specific value-based logics, which shape expectations, hopes, policies, designs and implementation related to POCTs, and structure the social relationships in which they are embedded. These logics include technological autonomy, scalability, care, rapidity and certainty. We propose that these evolving logics might be considered a key characteristic of the global health innovation ecosystem as it intersects with health systems. These value-based logics are perpetually renegotiated and transformed throughout the POCT life cycle, reconfiguring the dynamic relationships that constitute health systems (Figure 3). Taking these logics into account can contribute to reflexive and socially informed policies for the design and deployment of POCTs and for technological innovation in global health more broadly.

#### Technological autonomy

POCTs exemplify the contemporary orientation of global health innovation towards device-based solutions (Biehl and Petryna, 2013; Reubi, 2016; Street, 2018). Across spaces of global health, international development and humanitarianism, scholars have described the growing prominence of ‘small technologies of government’, little devices that are designed to be portable, inexpensive and scalable and have increasingly become the focus of international efforts to foster social and economic improvement (Collier *et al*., 2017). ‘Based on the use of biomedical technologies to eliminate diseases one at a time’, device-centric thinking articulates the decline of ‘universalist’ approaches to health systems strengthening in favour of a model of ‘access’ to life-saving technologies (Kelly and Beisel, 2011; Packard, 2016, p. 270).

In line with this scholarship, many of the papers we reviewed drew attention to the technological optimism invested in the POCT device by funders, policymakers, regulators and public health professionals (Hutchinson *et al*., 2015; 2017b; Beisel *et al*., 2016; Umlauf, 2017; Umlauf and Park, 2018; Janssen *et al*., 2021; Beckmann *et al*., 2022; see also Haenssgen *et al*. (2018) for an example of such initiatives for antimicrobial stewardship). The central promise of POCTs is the capacity to bypass reliance on existing weak infrastructure; POCTs offer an ‘autonomous determining force’ as Hutchinson *et al*. (2017b) describe, which will ‘enable swift diagnosis, without the necessity of significant investment in training, equipment or infrastructure associated with the development of laboratories’ (Hutchinson *et al*., 2017b, pp. 1077–78).

Underpinning these expectations of technological autonomy are often implicit, ahistorical imaginaries of existing health systems as naturalized sites of lack, absence and state failure (Engel, 2020; Street, 2023). Collectively, these studies demonstrate that throughout their social lives, POCT technologies interact with health systems in complex and various ways that depart significantly from an autonomous model of technology. While health system infrastructures, including medical expertise, organizational capacity and diagnosis and treatment options (Beisel *et al*., 2016), may be described as ‘fragmented’, ‘unstable’ (Engel *et al*., 2017), ‘incomplete’ (Umlauf, 2017) and ‘under-equipped’ (Beisel *et al*., 2016), this body of work tends to highlight the thick socio-material relationships through which these purportedly ‘under-resourced’ health systems work.

Focusing on a public health emergency of international concern, Kelly *et al*. (2022) demonstrate the significance of those embedded (and often invisibilized) relationships in containing an outbreak, and the implications of supressing their *ad hoc* capacities to the logics of autonomy that drive the global health emergency response. They describe how, in development of Ebola rapid diagnostic tests (RDTs), little consideration was given to existing waste management infrastructure in community health facilities, an oversight that ultimately contributed to the use of Ebola RDTs being limited to laboratory settings during the outbreak. Similarly, in Uganda, Umlauf and Park describe how unreliable supply chains—the infrastructures that move health resources within the Ugandan health system—result in regular malaria rapid diagnostic test (mRDT) stock-outs in primary healthcare facilities, rendering them unreliable infrastructures themselves. Far from operating as autonomous devices, mRDT use in this setting was contingent on the infrastructures and medical expertise they were imagined to replace.

Interactions between health workers and POCT devices in under-resourced settings are especially instructive for helping to reimagine technology–system dynamics. Many of the papers emphasize the ‘severely limited resources’ (Beckmann *et al*., 2022), and ‘high-pressured’ environments, within which health workers are expected to diagnose and treat patients (Beisel *et al*., 2016; Engel *et al*., 2017). The papers describe how expectations that POCTs will streamline work in such contexts are not borne out in the experiences of health workers, who find that POCTs add to their already extensive workloads. For example, in Uganda, Beisel *et al*. (2016) describe the processes that surround testing with mRDTs, including administering the test, waiting for results, pre-test and post-test counselling, and inputting results into health information systems (see also Engel *et al*., 2017; Beckmann *et al*., 2022). Collectively, the body of papers showed that there is never any moment in which these technologies might be considered as standing apart from or acting outside existing health systems. Rather, the use of POCTs depends on the ability of an already overstretched workforce to accommodate new technologies and to reconfigure and rebuild the health system around them.

While most of the studies focus on the public health sector, some also address commercial actors and interests as important features of health systems (Hutchinson *et al*., 2015; Engel *et al*., 2017; Haenssgen *et al*., 2018; Kelly *et al*., 2022; Street, 2023), showing, for example, the decentralized and minimally regulated use of biomedical devices and materials (including medicines) by local commercial actors (Hutchinson *et al*., 2015; Engel *et al*., 2017; Haenssgen *et al*., 2018). In the examination of the introduction of mRDTs through drug shop vendors in Uganda, Hutchinson *et al*. (2015) illuminate how the uptake of mRDTs within localized market logics meant that drug shop vendors leveraged these devices to signal their expertise and augment the profitability of the drug shop. As in public settings, in these private settings, the use and meanings of mRDTs were conditioned by the context and logics in which they were embedded.

The version of the health system that comes into view when following the social lives of POCTs is not a coherent system of ‘building blocks’ (World Health Organization, 2010), or a set of identifiable bounded parts, as health systems are often conceptualized in policy-based literature and research (Kielmann *et al*., 2022). Instead, it provides insight into the dynamic interrelationships between people, technologies and infrastructures that co-constitute health care, within which people constantly negotiate the value of practices, objects and expertise. POCTs often reconfigure relationships within those systems in profound ways, but they are best conceptualized as constitutive elements of health systems rather than autonomous devices that circumvent them. The social lives of POCTs demonstrate the limitations of autonomous models of technology, while at the same time showing that technologies have the potential to transform health system relationships and practices in positive ways. Health workers who participated in the research reviewed here did not value POCTs as autonomous technologies, but that is not to say the technologies held no value for them at all. Instead, the value of POCTs emerged from what was possible to do and achieve with them within existing health system relationships.

#### Care

Within global health innovation frameworks, POCTs are often imagined as caring commodities. Their very existence in a marketplace, otherwise characterized by a pervasive lack of industry interest and investment, is perceived to depend upon a proprietary innovation ecosystem in which manufacturers retain intellectual property (IP) and investors and developers recoup initial investments. In this view, POCTs are necessarily developed as products, designed to be sold to governments, private health companies and donors in a global health marketplace. Market-based innovation frameworks have been subjected to extensive critique in critical studies of global health (Biehl and Petryna, 2013; Erikson, 2015; Mcgoey, 2021). In these critiques, valuations of markets and commodities are often opposed to those of care and people. However, the papers reviewed here demonstrate the extent to which care is also a foundational value in the efforts by global health actors, including start-up entrepreneurs, developers, policymakers and regulators, to stimulate diagnostic innovation and orchestrate the dissemination and use of diagnostic tools.

Street describes how two seemingly opposed value frames, the value of life undergirding humanitarian concern and the value of commodities foundational to capitalist systems, were negotiated under the umbrella of ‘humanitarian entrepreneurship’ in the effort to develop and manufacture a CD4 testing device for HIV treatment as a ‘commodit[y] that care[s]’ in Boston, USA. However, a potential market for this device hinged on the adjacent values of care that underpin global health standard setting and which are guided by ‘a commitment to progressive improvement in science, technology and practice’ (Street, 2023, p. 12). When mounting clinical evidence in the USA suggested viral load testing to guide HIV treatment as superior to CD4 testing, the WHO abandoned recommendations that CD4 count guides HIV treatment. This policy shift, upholding a standard of healthcare universalism based on ‘evidence’ over a pragmatic approach reflecting actual diagnostic landscapes, meant that innovators’ target market of donors and the potential commodity value of the device evaporated. This case study illustrates the tensions and negotiations involved in defining and designing care among global health actors, and the forms of care that may (or may not) materialize.

In contexts of use, POCTs can similarly come to sit at the interface between different logics of care. Beckmann *et al*. (2022) illuminate the challenges health service providers face in balancing the values of ‘body work’ demanded by clinical encounters and the values of laboratory work, demanded by use of HIV RDTs. While the clinical encounter called for care infused with compassion, good rapport and emotional engagement, laboratory values inscribed in RDTs required care underpinned by emotional detachment from the person, physical distance and zooming in on body parts and samples rather than an engagement with the whole person. Health service providers struggled to commensurate the demands of these care value frames when testing patients using the HIV RDT. The authors conclude that the balancing act of these value-frames results in ‘messiness’, which expanded misdiagnosis of HIV.

The logic of care that underpins efforts to develop and deploy POCTs often hinges on the view that good care should be standardized and evidence based (Parker and Harper, 2006; Adams, 2013). Within this logic, the POCT will improve care because it circumvents the agency of the individual health worker to render practices of care uniform. Several papers describe how health personnel find themselves caught between this logic of care, which abstracts care from relationships (Adams, 2013), and existing understandings of care in their place of use, as premised on individualized clinical relationships between the patient and caregiver. Beisel *et al*. (2016) demonstrate how mediation between these logics of care, ‘requires delicate moral negotiations of health care staff between treatment guidelines [based on mRDT results] and the suffering patients’ (p. 4). Such moral negotiations are especially fraught when the purpose of the POCT is to withhold medication from those who test negative for a disease and when expecting that treatment be based solely on POCT results therefore equated to withholding the only care available to sick patients (Beisel *et al*., 2016; Hutchinson *et al*., 2017b; Haenssgen *et al*., 2018). Umlauf (2017) and Umlauf and Park (2018) similarly describe how basing malaria treatment decisions on the mRDTs required health service providers in Uganda to prioritize biomedical disease knowledge over ‘empathic care’, which recognizes the socio-economic conditions of patients and moments beyond the immediate disease episode.

Through their use of POCTs, these papers show that healthcare providers navigated competing care logics, balancing the values of standardization, stewardship and evidence-based medicine with their moral obligations to sick patients. As a whole, these papers illuminate the multiple ideas of care inscribed in POCTs, from the notion of commodities that care to that of ‘evidence-based’ care. However, these understandings of care are constantly negotiated and transformed in relation to other ideas of care, such as those intrinsic to the social work of clinical encounters, throughout processes of POCT innovation and use.

#### Scalability

The synthesized papers capture the expectations of scalability embedded in POCT development and deployment, that is, the portrayal of their transformational promise as deriving from their ability to reach and standardize diagnosis and treatment pathways globally (Haenssgen *et al*., 2018; Beckmann *et al*., 2022). By virtue of their supposed ease of mobility, POCTs render imaginaries of diagnostic standardization possible even in the absence of laboratory systems (Beisel *et al*., 2016; Kelly *et al*., 2022; Street, 2023), making POCTs universally usable across diverse settings and people, ‘simplifying the procedures on site and … at the same time standardizing between locales’ (Engel and Krumeich, 2020, p. 2). The reviewed papers revealed the centrality of fantasies of universal reach and standardization to the global health imaginary, as well as the extent to which ‘little devices’ (Collier *et al*., 2017) like the POCT have become key governmental technologies in the ‘global health complex’ (Mcgoey *et al*., 2011). What is being scaled, in other words, when POCTs are distributed to primary health centres globally, is not only diagnostic access but also relationships of authority, expertise and control. In this respect, POCTs might be viewed as technologies for extending the reach of the state (or global governing actors) to the health system periphery (Biehl, 2005).

However, the papers show that the scalability of POCTs cannot be taken for granted. The achievement of scale, both in terms of mobility and standardization, requires significant effort across the life cycle of the device and POCTs frequently fail to deliver on this promise. Mobility, for example, is not a given characteristic of the POCT but needs to be actively engineered, maintained and controlled. Street describes how developers of a CD4 testing device prioritized ‘robustness’ as a design feature within the development process, spending months ensuring that the device would have thermostability at high temperatures and not be damaged during transportation. The story of this particular device, however, also revealed some of the social and technical challenges involved in achieving geographic scale, with the efforts to engineer thermostability coming into tension with the valuations of investors, ultimately pushing the entire start-up company into risky financial waters.

Moreover, post-development, mobility not only allows POCTs to expand reach but can also enable unanticipated diversions. Umlauf and Park describe how mRDTs procured through the public health system in Uganda were intended for primary-level health facilities without access to laboratory-based microscopy diagnosis of malaria. However, thousands of these portable devices were diverted during each procurement cycle to government hospital laboratories, where they were desired for their rapidity of diagnosis, compared with microscopy, and to compensate for the perceived poor quality of microscopes. This meant that less than half of the planned number of tests landed in primary health facilities, contributing to the normalization of stock-outs discussed under the ‘Technological autonomy’ section. Thus, while their mobility has the potential to expand access to diagnostic services, it can also contribute to exacerbating long-standing inequities in access to diagnostic services.

Other papers showed that the deployment of POCTs as technologies of governance required the production of new subjectivities. Hutchinson *et al*. (2017b) describe the introduction of mRDTs to guide the prescription of antimalarials in public health facilities in Tanzania. The training that personnel received, they argue ‘presented the mRDT as a representation of modern biomedicine and those who used it as modern clinicians in opposition to traditional doctors who continued to use signs and symptoms’ (p. 1083). As new, ‘modern’ doctors, clinicians were expected to align their clinical practice to the standardized procedures of the state. This involved demanding new relationships between providers and patients, as providers were expected to base decisions for prescribing antimalarials solely on the results of the mRDT, rather than on the human interaction in the clinical encounter. In practice, however, health service providers only partially assumed these new subjectivities. ‘Health workers, while keen to use the test, also incorporated concerns about patients who failed to recover after several visits to the health facility, patient’s interpretations of their illness, clinical signs and symptoms and a history of patient’s movement’ (p. 1083), as well as considerations of the socio-economic status, when making decisions related to antimalarial prescription. These papers illuminate social relationships as foundational to diagnostic processes. While POCTs reconfigure these relationships, they do not single-handedly generate uniformity in use, as use is shaped by a multiplicity of considerations within these relationships.

The synthesized papers reveal the extent to which nonstandardized practices of ‘creative work’ (Beisel *et al*., 2016; Hutchinson *et al*., 2017b), ‘improvization’, ‘tinkering’ (Engel *et al*., 2017; Engel, 2020; Beckmann *et al*., 2022) and ‘coordination’ (Engel *et al*., 2017) are often necessary to make POCTs work in the midst of multiple, shifting and competing demands. The results in variation across POCT processes, diagnoses and treatment pathways throughout the life cycle are therefore best conceptualized as essential to this ‘fluid technology’ (De Laet and Mol, 2000; Engel *et al*., 2017) rather than as a dangerous aberration.

#### Rapidity

In global health logics, POCTs are commonly celebrated as a rapid replacement to the slower work of laboratory-based diagnosis. However, several studies found that at the point of use, health workers were less likely to compare the time it took to use a POCT with the time-to-result of laboratory testing than to compare the time it took them to undertake a patient consultation before and after the introduction of the POCT (Beisel *et al*., 2016; Umlauf, 2017; Umlauf and Park, 2018; Beckmann *et al*., 2022). Within health facilities, POCTs are often experienced as slowing down processes of diagnosis when compared with the more rapid practices of clinical diagnosis, especially when this consists of a ‘light version of professional examination’ as Umlauf describes in Uganda (p. 456). Not only do POCTs take more time to perform and generate results than clinical diagnosis, they are often accompanied by expectations of counselling (Beckmann *et al*., 2022) and documentation (Beisel *et al*., 2016), compounding the time demands of the test for health service providers. In such cases, POCTs are not experienced as rapid but as slow and time-consuming.

To compensate for the added time pressures, Beckmann *et al*. (2022) and Beisel *et al*. (2016) write of tactical strategies employed by health service providers, including simultaneous counselling, testing, examination and data collection; conducting tests in batches; quick reading of results, i.e. reading results after 2–5 min rather than waiting the recommended 15 min; and falling back to clinical examination, even when tests were available. Although such strategies were viewed as justifiable by the overstretched health service providers, the authors identified these as potentially compromising the validity of the tests and the interpretation of test results.

The time demanded by POCTs was generally viewed as a disadvantage in public health facilities in these studies. In contrast, Beisel *et al*. (2016) found the additional time implications of mRDT use in drug shops in Uganda to be an advantage for drug shop vendors. The possibility of accessing the test in a nearby drug shop meant that people saved time by avoiding travel to the public health facility for a diagnosis. Moreover, it extended the time of attention in encounters between patients and drug vendors, strengthening these relationships. This worked to the advantage of drug shop vendors, whose clientele increased and thereby the profitability of drug shops.

Furthermore, while expectations of POCT rapidity often focus on the turn-around time for test results, several studies showed the diagnostic process to extend beyond and after the moment of testing. In South Africa, Janssen *et al*. (2021) describe how HIV status is constructed through consecutive testing moments, the duration and syncopation of the lived experience of health and illness, and knowledge of one’s engagement (or not) in risk behaviours across time. The authors argue that:

A test result might appear in an instant, but knowing one’s status isn’t a moment—it’s an ongoing process of viewing test results, considering your behaviours and the behaviours of those around you, observing the things your body is doing, keeping track of how long it’s been since you last tested, and knowing when you should test again. Even with a positive result comes the next step of confirmatory testing and then pre-treatment counselling. (Janssen *et al*., 2021, p. 16)

Similarly, in South India, Engel *et al*. describe diagnosis as dispersed across various health actors and settings, with people moving back and forth between public and private providers to achieve diagnosis. Most of the work to coordinate between these actors and spaces falls to patients, who engage in ‘coordination’ work between them to arrive at diagnosis. The authors write that ‘presumed test and treat cycles are often not circular or linear but messy, intricate with overlaps, detours, bypasses, and frictions, frustrations and competitions in-between the different steps’ (p. 67). These studies demonstrate that rather than ‘rapid’ technologies that deliver a definitive diagnosis at the point of care, POCTs are part of longer, fragmented journeys towards diagnosis that are distributed across time and space.

The reviewed papers showed how logics of rapidity intersected with other temporal logics in global health. During moments of crisis or emergency, for example, Kelly *et al*. show that the logic of ‘rapid’ testing can become associated with a logic of ‘accelerated’ product development. However, the tempos of this ‘accelerationist’ paradigm are often mismatched with the tempos of existing global health research and development. In the case of the Ebola and Zika epidemics, this mismatch not only slowed the deployment of devices, but prioritization of rapid testing technologies also resulted in the innovation of devices that lacked clear purpose and were often inaccurate. Temporal practices across these differing tempos meant that the promise of rapid scalability of POCTs to respond to these epidemics was never fully realized.

The issue of ‘alignment’ or ‘synchronisation’ of different tempos within POCT development and deployment was also explored in papers by Engel and Street. In the innovation of HIV and tuberculosis (TB) POCTs, Engel describes the multiple timescales that come into play, the alignment of which is an achievement. Business and funding timescales must align with those of development and manufacturing, and market entry must align with the timescales of infrastructure development plans and procurement in countries. These tempos of innovation must also align with expectations and needs at the point of care, which may require revisiting device design after introduction in these spaces. For example, in developing and deploying a CD4 device, developers found that Zimbabwean farmers required a differently sized lancet than that built into the device, due to their thick calluses. Developers therefore had to return to the design and integrate a new lancet and sample volume, thus slowing down the deployment of the device. Engel concludes that the ‘temporal dimension of alignment work, continuously ongoing and across development steps, means that alignment is only ever stabilized temporarily, and one cannot easily draw a line to pinpoint a well-aligned global health diagnostic’. In a similar vein, Street develops the concept of ‘synchronization’ to conceptualize the temporal work of aligning distinct value regimes in POCT innovation and development. In the case of the design of the CD4 POCT for guiding HIV treatment, she describes the misalignment between the tempos of start-up investment entities that provide financial resources to entrepreneurs designing devices based on fixed timescales and those of technology developers.

Across the product life cycle, the reviewed papers show that the temporality of the POCT is constructed and negotiated within multiple distinct temporal registers, from the cyclical tempos of start-up investment and R&D to the punctuated immediacy of emergency and crisis, to the routine time pressures of everyday clinical work in under-resourced settings. POCTs are revealed to be complex temporal objects that can either speed up or slow down experiences of diagnosis and innovation, depending on the wider constellation of relationships and practices within which they are embedded.

#### Certainty

Within global health logics, POCTs are often valued as epistemic tools that can dispel diagnostic uncertainty associated with clinical diagnosis and authoritatively inform clinical and public health decisions. As such, POCTs are intended to extend the authoritative biomedical knowledge practices of the laboratory beyond the walls of the laboratory (Street, 2023).

Engel and Krumeich (2020) identify accuracy—the ability of a test to generate certainty evaluated in terms of sensitivity, i.e. correctly identifying the presence of a health condition, and specificity, i.e. correctly identifying the absence of a health condition—as the primary means of evaluating the certainty that a POCT can help to achieve in global health settings. However, in POCT innovation, developers must negotiate the value of accuracy alongside other values, and trade-offs to improve accuracy often result in the test having reduced reach. In designing TB tests, the authors show that prioritizing accuracy meant prioritizing the Xpert MTB/RIF, a highly accurate but expensive and infrastructure-dependent test. Developers reasoned that the high accuracy of the test would allow it to be more profitable as it could be marketed in high-income settings; however, in so doing, it set an accuracy benchmark that other less expensive and less operationally constrained tests—tests that would be more accessible in LMICs—could not attain, therefore precluding their development.

Uncertainty over the test purpose also looms over questions about the diagnostic certainty a test can provide, as discussed by Kelly *et al*. (2022) in the context of epidemic emergencies. This was especially evident in the development of a test for Zika virus, which posed the greatest risks when it infected pregnant women, as it can cause microcephaly in the foetus. While the purpose of POCTs for mapping Zika was evident for a variety of global health actors, the clinical-use value was less clear. During the Zika epidemic response, not only were treatment options unavailable, but for women diagnosed with Zika, the possibility for pregnancy termination was largely unavailable given the legal proscription of termination in Brazil. ‘As a result,’ the authors write, ‘the definition of a clinical use-case for new diagnostic tools was directly entangled with local struggles over reproductive health and rights, and the politically charged issue of how new diagnostics would be linked to concrete interventions reverberated throughout the WHO-sponsored product development process’ (p. 197). This uncertainty of purpose contributed to the fact that by the end of the epidemic, only two POCTs for Zika were included in the diagnostic procurement list.

Furthermore, along the POCT life cycle, the synthesized studies illuminate POCTs as epistemic tools negotiated within a variety of knowledge practices to generate diagnostic certainty in clinical practice. Beckmann *et al*. (2022) describe health service providers in Zimbabwe balancing HIV RDT test results with their embodied knowledge of HIV based on their own experiences with the virus and encounters with patients. When the test results and embodied knowledge aligned, providers confirmed diagnosis. In the case of discordant results, however, providers did not simply accept the POCT results over their embodied knowledge. In such cases, POCT results contributed to uncertainty as health service providers were suspicious of the test results, leading them to engage in further knowledge practices such as consulting with colleagues and advising follow-up tests to arrive at diagnosis.

Other studies considered the negotiation of POCT results with practical (Hutchinson *et al*., 2017b; Janssen *et al*., 2021), situated (Umlauf, 2017) and locally produced knowledge practices (Hutchinson *et al*., 2017b; Haenssgen *et al*., 2018). Haenssgen *et al*. (2018) explored the historical and social, locally produced constructs of antibiotic use in Thailand, within which C-reactive protein (CRP)-POCTs were introduced as epistemic devices in public health facilities. These were used to detect CRP in the bloodstream of patients experiencing fever, indicating the beneficial use of antibiotics, or, alternatively, demanding the withholding of antibiotics in the case of a negative result. In this context, however, health service providers balanced POCT results with their own expert judgement to determine the necessity of antibiotics. Some health service providers considered the CRP-POCT equivalent to ‘laboratory testing’, which trumped their own judgement by conclusively indicating that, as one nurse expressed, ‘there’s got to be something wrong somewhere in the body’ (p. 8). In other cases, health service providers’ ‘own judgement’ of the need for antibiotics was used to override the POCT results.

Related to issues of certainty, three papers considered trust in the POCT device (Hutchinson *et al*., 2017a; Haenssgen *et al*., 2018; Janssen *et al*., 2021). In using HIV self-tests in South African clinics, Janssen *et al*. (2021) found that the visibility of the test results served to instil confidence is the test results as ‘authentic and correct’. Visibility was also crucial to trust in CRP-POCT testing in Thailand, where Haenssgen *et al*. (2018) found that health providers used the visibility of test results to encourage patients to accept the withholding of antibiotics as the correct treatment.

However, these researchers also found that visibility of test results alone was insufficient for generating trust. Janssen *et al*. (2021) and Haenssgen *et al*. (2018) consider how bodily samples figured in the trust people had in the POCT device. Janssen *et al*. (2021) found that people were less trusting in the HIV self-test based on the test format’s reliance on saliva rather than blood. This was in part because they were accustomed to HIV testing through blood samples and their association of blood with the transmission of HIV; therefore, a saliva-based test was experienced as antithetical to this knowledge. The use of blood also instilled confidence among patients in Thailand using the CRP-POCT. This trust was perhaps overextended, as people interpreted the CRP-POCT as a comprehensive blood test and the negative presence of CRP in the bloodstream as an overall indication of good health (Haenssgen *et al*., 2018).

Throughout these papers, the value of POCTs as epistemic tools and objects of trust was negotiated in relation to multiple historically and socially situated knowledge practices and relationships with testing technologies. Despite expectations for POCTs to extend the authority of the laboratory in arriving at diagnostic certainty, which are often found during moments of policy-making, investment and product development, these devices do not inevitably replace other knowledge practices but are embedded and negotiated within them. Moreover, rather than dispelling uncertainty by offering a conclusive diagnosis in the form of a binary test result, POCTs can contribute to new forms of uncertainty. These papers point to knowledge practices as multiple, and POCTs as contributing to, rather than reducing, this multiplicity.

## Discussion

In offering a compact, mobile and ostensibly simple solution to global inequities in access to diagnostic services, POCTs have emerged as an archetypal global health technology (Street, 2018; Pai *et al*., 2019; Fleming *et al*., 2021). The priority status of diagnostics in global health is now widely recognized, and their imagined purpose is greatly expanded beyond that of contributing to vertical disease programmes for high-profile diseases (Moussy *et al*., 2018; Fleming *et al*., 2021). The publication of the WHO’s Essential Diagnostic List highlighted the importance of the availability of a whole suite of tests to support routine primary care (World Health Organization, 2019); campaigns for neglected tropical diseases have highlighted the importance of diagnostics to goals of disease elimination as well as to improvements in the efficacy and efficiency of therapeutic care (Taylor, 2020; World Health Organization, 2020); and the widespread use of self-testing during COVID-19 has opened up imaginaries of decentralized diagnostic systems that are non-reliant not only on laboratories but also on professionalized care (García-bernalt Diego *et al*., 2022; Budd *et al*., 2023; Titanji and Pai, 2023).

As diagnostic systems are reimagined and reinvented, the findings of this meta-ethnography offer insight into the interplay between technological innovation and health systems. A social lives approach helps to overcome conceptual divisions between ‘upstream’ and ‘downstream’ or between innovation and deployment in our imaginaries of global health. Instead, our findings show that innovation and health systems are entangled across the life course of medical technologies. The value-based logics that shape funding priorities, regulation, R&D processes and policy-making are often premised on assumptions about what health systems are and how they work, which turn out to be unrealistic in the face of the dynamic relationships between infrastructures, bureaucracies, technologies and people that constitute health systems in practice (Kielmann *et al*., 2022). Tempering technological expectations to the everyday realities of health system work and facilitating greater involvement of users in policy and product development could have far-reaching benefits for the effective development and design of POCT devices in the future. This may, for example, entail a consideration of the realistic impact of POCT devices on existing workflows and burdens in under-resourced health systems and how POCT design and protocols might minimize this. It may involve an appreciation for the limitations of POCT devices as technologies of care within existing expectations and logics of what care entails in places of use. It may lead to greater understanding of the contribution that POCTs can make to diagnostic certainty once diagnosis is recognized as a spatially and temporally distributed process. None of these considerations undermines the value and utility of the POCT device, but it recognizes that an in-depth understanding of health systems as social systems is necessary to ensure the product retains value for people across its life cycle.

If a social lives approach to technologies requires that we rethink our conceptual models of health systems, and our assumptions about technologies as having an autonomous status external to those systems, then it also requires that we reconsider what we mean by innovation and where we locate it. Our synthesis shows that imaginaries of health systems are always already present at stages of policy, regulation and R&D. Conversely, it shows that innovation does not end with taking a product to market but is also an essential quality of health systems and people’s efforts to make novel devices work in complex scenarios (Hyysalo *et al*., 2019). Understanding and recognizing this creative work, while also acknowledging that it can sometimes be an unnecessary additional burden for the people that use and work in health systems, is important for expanding our imaginaries of the purposes to which technologies might be put and the kinds of value they might hold for their intended (and unintended) users.

To some extent, a health-systems approach to global health innovation has already become established in the field of global health diagnostics. This is evident, for example, in the shift in strategy at Foundation for Innovative New Diagnostics (FIND), from a focus on the standalone device to a focus on how diagnostic technologies can contribute to universal health coverage (FIND, 2015), and the recent World Health Assembly declaration on strengthening diagnostic capacity, which goes beyond a focus on technological innovation to emphasize the ‘systems and resources needed to ensure everyone who needs a test can get one’ (World Health Organization, 2023). Yet even as diagnostic imaginaries appear to be expanding to incorporate commitments to health systems strengthening beyond the technical fix (Pai and Kohli, 2019; Pai *et al*., 2019; Kao *et al*., 2023), the current ‘austere’ climate of global health funding (Kentikelenis and Stubbs, 2022; Stubbs *et al*., 2023) augurs possible imaginative foreclosures on the horizon. With a global recession looming, and international resources for health in LMICs unlikely to improve in the near future (Kentikelenis and Stubbs, 2022), the possibility that POCT imaginaries may contract back to an autonomous model of stand-alone technology haunts the current innovation ecosystem.

Within this context, the development of holistic approaches for the evaluation and review of technologies, which go beyond narrow, often quantitative, assessments of ‘acceptability’ to understand the relationships between persons, infrastructures and technologies involved in health system transformation, is crucial. We suggest that meta-ethnography offers a possible valuable approach to evaluation and accountability, enabling us to take stock of a field of policy and intervention and to understand the impact it has had on people and systems in different settings and across a long time period, thereby providing a kind of anthropological counterpart to the metrics-driven domains of impact evaluation and implementation research (Strathern, 2000; Adams, 2013; 2016), which make minimal use of social science theory and qualitative evidence (Van Belle *et al*., 2017; Kislov *et al*., 2019).

We suggest that meta-ethnography also holds untapped potential for contributing to new theories and ideas in the social sciences. While meta-ethnography was developed within the social sciences (Noblit and Hare, 1988), the meta-ethnography revival over the past two decades has occurred predominantly outside the social sciences and in the field of health sciences (Booth, 2019; France *et al*., 2019b). The regeneration of meta-ethnography in this field was motivated by a desire within global health and the health sciences to better engage with qualitative data and to provide a qualitative answer to the well-established fields of systematic review and meta-analysis, which have come to dominate these disciplines as providing authoritative knowledge in the spirit of ‘evidence-based practice’ (Lewin and Glenton, 2018; Downe *et al*., 2019; Glenton *et al*., 2019; Lewin *et al*., 2019). This work provides valuable contributions for making meta-ethnography a viable approach for engaging with qualitative evidence across disciplines, and equally signals an important turn in valuing qualitative evidence, long marginalized within global health (Green, 2014; Greenhalgh *et al*., 2016; Loder *et al*., 2016; Stickley *et al*., 2022). However, we suggest that meta-ethnography opens promising pathways within the social sciences, as well, for example contributing to the theorization of technological innovation and health systems as socially entwined fields of value-generation. It can also open new pathways for guiding social science enquiry, for example, we see each of the logics emerging through this synthesis as meriting further investigation. Such approaches have the potential to expand the contributions of social science across disciplinary boundaries and uptake in global and national health policy-making.

### Strengths and limitations

A strength of our meta-ethnography is that it was led by a team of social anthropologists. While this type of work is uncommon within anthropology, we consider this a strength as meta-ethnography is rooted in the non-positivist research epistemologies, such as interpretivism, constructivism and critical theory. These epistemologies are first nature to the research team, who were well positioned to engage both with the approach and with the evidence synthesized.

One of the shortfalls of social science publishing is that it rarely operates within a responsive timeframe. There is, therefore, a time lag between the observations made in the papers reviewed and current policy and practice related to POCTs. In the years since the first paper that we reviewed was published, in 2011, the role imagined for POCTs has also changed dramatically, with funders, policymakers and public health experts increasingly shifting away from a focus on their role as standalone technological solutions within vertical disease programmes towards an emphasis on the importance of diagnostics for achieving universal health coverage and contributing to health systems strengthening (Moussy *et al*., 2018; Pai and Kohli, 2019). Our study is also impacted by this time-lag and may not account for the most recent shifts in response to global health austerity in a post-COVID-19 context (Stubbs *et al*., 2023). Moreover, we were unable to provide a complete account of the POCT life cycle through the synthesized papers. A particularly glaring absence is that of waste management, which tends to be marginalized in debates related to diagnostics and more generally in global health; however, we would like to signal this as an important area for future exploration, given its importance, particularly in LMICs where waste management in general tends to be challenging (Ongaro *et al*., 2022; Street *et al*., 2022).

## Conclusion

We identified five value-based logics that emerged out of social science research conducted across diverse country and health system settings and in relation to different disease programmes and testing technologies. Together, we suggest these logics might be understood as defining and motivating a technology-focused framework of global health innovation.

### Technological autonomy

These papers demonstrate the aspirations inscribed in POCTs as devices capable of circumventing systems characterized as weak, fragmented and generally lacking. However, across these accounts, devices do not circumvent unreliable systems and infrastructures; instead, they are incorporated within them, depend on them and act themselves as unreliable infrastructural components within them.

### Care

POCTs are often imagined as commodities that care, i.e. objects that can leverage market-based logics to expand the provision of healthcare service, and are capable of delivering ‘evidence-based’ treatment. However, what counts as good care, and how POCTs might contribute to its practice, is negotiated and transformed throughout the life cycle. At the point of use, understandings of care as standardized practice often come into tension with relational understandings of care.

### Scalability

Valued for their mobility and simplicity of use, POCTs promise to standardize diagnosis across scales, extending the reach of the state and global health actors. However, the project towards sameness is never complete, and is even an impossible goal, given the always necessary, unstandardized work undertaken by human actors to make POCTs work within the constraints and realities of real-world conditions.

### Rapidity

POCTs are often heralded for their potential to speed up the testing process. Logics of rapidity often also intersect with accelerationist paradigms of innovation. In contrast to these aspirations for speed, the innovation, deployment and use of POCTs can be experienced as either rapid or sluggish depending on the alignment of multiple tempos and temporalities.

### Certainty

POCTs are often imagined to carry the authoritative knowledge of the laboratory into diverse settings to enable health providers and patients to arrive at diagnostic certainty. However, certainty is both an unstable value—often requiring trade-offs in other values, such as access—and an often unachievable ideal. As they move into settings of use, POCTs both encounter multiple ways of knowing and often introduce new uncertainties.

Opening POCTs up to critical scrutiny in this manner allows us to render the values that underpin this innovation framework and its aspirations visible. We suggest that these values and aspirations matter, as they set the terms for what is desirable and possible and foreclose other imaginaries. A social lives approach shows that these values and logics are never settled, but they are always contested and negotiated within social relationships and across the life cycle of a technology. This synthesis, therefore, simultaneously illuminates and unsettles these logics, opening up the opportunity for thinking of global health otherwise (Adams *et al*., 2019; Street, 2023). We suggest that opening up a discussion around what POCTs are for and what they can achieve can contribute to more reflexive approaches to technological design, deployment and policy-making that understand innovation and health systems to be entwined and take into account the fluid values and relationships that shape the life cycle of a product. Such an approach is especially important in the present moment, in which diagnostic imaginaries are dramatically expanding, and yet the resources to implement these imagined futures are potentially contracting.

## Supplementary Material

czae054_Supp

## Data Availability

The full search strategies and datasets generated and analysed during the current study will be made available on the Edinburgh DataShare digital repository. The protocol for this study is registered with Prospero, ID number: CRD42022366518. It can be accessed at https://www.crd.york.ac.uk/prospero/display_record.php?ID=CRD42022366518.

## Reflexivity statement

The authorship of this paper comprises women and a mix of junior (J.P.) and senior (A.S., C.C. and A.K.) academics. These authors are all based in the UK. While including an author from an LMIC for this paper is not necessary as per the *Health Policy and Planning* guidelines since this is a review of published papers, we recognize that the authorship influences the review and interpretations drawn from it. In the systematic evidence mapping from which the papers for this review were drawn, we aimed to be inclusive by not placing limitations on language and by enlisting the support of colleagues who could assist in reviewing non-English papers. However, none of the non-English papers met the criteria for inclusion in this review.
