# Supplementary material for: The social lives of point-of-care tests in low- and middle-income countries: a meta-ethnography
Source: Health Policy Plan. 2024 Jun 22;39(7):782–98. doi: 10.1093/heapol/czae054 (PMC11308614; doi:10.1093/heapol/czae054)
Supplement: czae054_Supp [file czae054_supp.zip › suppl_data/Supplementary material 1_Search strategy.docx]

Supplementary material

Contents

[Appendix 1. Search strategy 2](#_Toc140762669)

[1.1 Inclusion and exclusion criteria 2](#_Toc140762670)

[1.2 Screening 3](#_Toc140762671)

[Appendix 2. Data Richness Scoring 4](#_Toc140762672)

# Appendix 1. Search strategy

For our exhaustive search, we included a broad range of terms and keywords related to three concepts: ‘point-of-care testing’, ‘qualitative research’, and ‘low- and middle-income countries’.

We identified the following key databases for our search to optimise the identification of articles which meet our inclusion criteria:

- MEDLINE (American health database)
- Embase (European health database)
- Anthropology Plus
- Web of Science (citation tracking index)
- CINAHL (Nursing)
- Scopus
- Global Index Medicus, World Health Organization
- Global Health (CABI)
- ProQuest Dissertations and Theses Global Database

In a second round of the search, we reviewed the references and citations of all the studies which met the inclusion criteria in the Web of Science to identify studies that were not picked up during the initial round. We also hand searched books and journals that were less likely to be picked up in the search.

All data bases were searched between 1 September 2022 and 8 October 2022.

## 1.1 Inclusion and exclusion criteria

See Table 1 for detailed inclusion/exclusion criteria

Table 1: Inclusion/exclusion criteria

| **Category** | **Inclusion** | **Exclusion** |
| --- | --- | --- |
| Publication date | After 2000 | Before 2000 |
| Type of evidence | Published studies; research studies; empirical studies;  grey literature in the form of theses and dissertations | Literature reviews; comment/opinion articles; pre-prints; grey literature in the form of reports and policy documents; abstracts and conference proceedings |
| Type of study (i.e. study design, methodology) | Qualitative studies (action research, case study analysis, discourse analysis, ethnography, focus group discussion-based, grounded theory, interview-based, narrative analysis, observation, participant observation, phenomenology); mixed-method studies where qualitative evidence is epistemologically distinct from and reported separately to quantitative evidence. | Quantitative studies; survey-based studies with open-ended questions; qualitative process evaluations; realist evaluations; mixed-method studies where qualitative evidence epistemologically resembles and/or is reported alongside quantitative evidence; study protocols |
| Environment | LMICs as context of deployment and use; research and development, regulatory, and policymaking settings based in high-income or LMIC environments that pertain to POCTs for use in LMICs | High-income countries as context of deployment and use; research and development, regulatory, and policymaking settings that pertain to POCTs for use in high-income countries primarily |
| Test format | In vitro POCTs (e.g., lateral flow immunoassays, molecular tests, nucleic acid amplification tests) | In vivo POCTs; urine-based pregnancy POCTs |
| Setting | POCT research and development settings; regulatory, policymaking, and programming settings; secondary health system settings; primary health system settings; community settings; non-formal health settings (e.g., drug shops); domestic settings | Laboratories (primarily) |
| Perspective | Any stakeholder perspective, e.g., those of POCT researchers and developers (scientists, academics), commercial actors (industry, manufacturers, salespeople) regulators, policymakers, health managers, formal health service providers, informal health service providers, patients | Lab technicians (primarily |
| Phenomena | Any social phenomena related to POCTs, either as a focus or peripherally | Social phenomena unrelated to POCTs, phenomena unrelated to social aspects |

## 1.2 Screening

Studies identified in the search were deduplicated. Using Covidence, each of the article identified through the search was independently screened by two team members. The full texts of articles which passed this phase were then retrieved, and eligibility for inclusion was independently assessed by two members of the team. Disagreements regarding inclusion or reason for exclusion were reviewed by a third team member and then resolved through discussion.

# Appendix 2. Data Richness Scoring

| **Score** | **Measure** | **Example** | **Look for** |
| --- | --- | --- | --- |
| 1 | Very little qualitative data presented; those findings that are presented are primarily descriptive | Action research or qualitative project evaluation where there is limited qualitative data presented | -Limited data presented from research participants -Very narrowly focused -Little to no interpretation, analysis |
| 2 | Qualitative presented; but narrowly focused and primarily descriptive | Qualitative programme evaluation with the presentation of descriptive qualitative data presented | -Narrow use of research methods -Narrowly focused on specific features -Minimal interpretation or analysis -Reveals little regarding the broader context |
| 3 | A reasonable amount of qualitative data presented; primarily descriptive | A typical qualitative research article in a journal, often embedded in a broader trial, is primarily descriptive, using simple thematic analysis | -Single or dual method -High degree of description -Thinner interpretation -Narrowly focused -Reveals some aspects of the broader context |
| 4 | A good amount and depth of qualitative data, conceptual analysis well-articulated | A qualitative research article in a journal with a larger word count that includes more context and setting descriptions and a more in-depth presentation of the findings | -Broader use of qualitative methods  -Deeper interpretations and analysis -Positions the research in relation to the broader context -Engages with social science theory |
| 5 | A large amount and depth of qualitative data; conceptual analysis well-articulated | From a detailed ethnography or a published qualitative article in a social science journal | -Rich presentation of qualitative data -Rich interpretation and analysis -Holistic account which reveals a great deal about the broader context and positions the research within it -Grounded in and builds on social science theory |
